# Effects of Rosiglitazone with Insulin Combination Therapy on Oxidative Stress and Lipid Profile in Left Ventricular Muscles of Diabetic Rats

**DOI:** 10.1155/2012/905683

**Published:** 2012-07-05

**Authors:** Servet Kavak, Lokman Ayaz, Mustafa Emre

**Affiliations:** ^1^Department of Biophysics, Faculty of Medicine, Yüzüncü Yıl University, 65100 Van, Turkey; ^2^Department of Biochemistry, Faculty of Medicine, Mersin University, 33343 Mersin, Turkey; ^3^Department of Biophysics, Faculty of Medicine, Çukurova University, 01330 Adana, Turkey

## Abstract

*Purpose*. In this study, we tested the hypothesis that rosiglitazone (RSG) with insulin is able to quench oxidative stress initiated by high glucose through prevention of NAD(P)H oxidase activation. *Methods and Materials*. Male albino Wistar rats were randomly divided into an untreated control group (C), a diabetic group (D) that was treated with a single intraperitoneal injection of streptozotocin (45 mgkg^−1^), and rosiglitazone group that was treated with RSG twice daily by gavage and insulin once daily by subcutaneous injection (group B). HbA1c and blood glucose levels in the circulation and malondialdehyde and 3-nitrotyrosine levels in left ventricular muscle were measured. *Result*. Treatment of D rats with group B resulted in a time-dependent decrease in blood glucose. We found that the lipid profile and HbA1c levels in group B reached the control group D rat values at the end of the treatment period. There was an increase in 3-nitrotyrosine levels in group D compared to group C. Malondialdehyde and 3-nitrotyrosine levels were found to be decreased in group B compared to group D (*P* < 0.05). *Conclusion*. Our data suggests that the treatment of diabetic rats with group B for 8 weeks may decrease the oxidative/nitrosative stress in left ventricular tissue of rats. Thus, in diabetes-related vascular diseases, group B treatment may be cardioprotective.

## 1. Introduction

 Hyperglycemia induces protein glycation, systemic low grade inflammation, and endothelial dysfunction [1]. As a consequence, diabetes is one of the main risk factor for cardiovascular disease. Hyperglycemia-induced endothelial dysfunction is characterized by an enhanced production of reactive oxygen species (ROS), which are important actors in the development of vascular damage. Consistently, antioxidant agents are able to rescue hyperglycemia-induced vascular dysfunction [1, 2].

 Insulin resistance is a fundamental abnormality in the pathogenesis of type-2 diabetes. A number of different mechanisms have been proposed to explain the mechanism of insulin resistance. Recent information suggests that a common feature of the development of insulin resistance is an increased production of ROS and that reduction in ROS production results in improved insulin sensitivity [3].

 Rosiglitazone, a member of the thiazolidinediones (TZD) class of antidiabetic agents, is an agonist of the nuclear hormone receptor peroxisome proliferator gamma (PPAR*γ*). Expression of these receptors is most abundant in adipose tissue where they play a central role in adipogenesis and lipid metabolism [4].

 Thiazolidinediones (TZD) are used clinically in diabetic patients by virtue of their insulin-sensitizing action, conveyed by the activation of the nuclear transcription factor PPAR*γ* [5]. In addition, these agents have remarkable pleiotropic activities: by improving endothelial function and systemic inflammation, they are expected to exert direct beneficial effects on cardiovascular risk, which are not mediated by the improvement in glucose metabolism. In this regard, pioglitazone was shown to abolish ROS production in 3T3-L1 adipocytes [6], whereas RSG reduced NADPH-stimulated superoxide production in aortas from diabetic mice [7], and troglitazone diminished ROS generation in leukocytes from obese subjects [8]. However, the molecular mechanism by which TZDs attenuate oxidative stress is not clear.

PPAR-*γ* activation reduced O^2−^ generation and NADPH oxidase expression in vascular endothelial cells in vitro and increased NO production through PPAR*γ*-dependent mechanisms [9].

In this study, treatment of insulin and rosiglitazone may decrease oxidative stress in diabetic rats, which may be cardioprotective in setting diabetic vascular disease, Therefore, the aim of the present study was to dissect the molecular mechanisms underlying the effects of RSG on hyperglycemia-induced ROS production.

## 2. Material and Method

### 2.1. Animal Handling and Treatment Protocol

Twenty-four healthy male Wistar albino male rats (250–320 g) were selected for the study. All animal procedures were performed according to the Guide for the Care and Use of Laboratory Animals of the US National Institutes of Health and approval of the ethics committee of our institution was obtained before the commencement of the study. The diabetic rat model used in our experiments was based on partial damage of pancreatic beta-cells resulting from a single administration of streptozotocin (45 mg/kg, STZ, Sigma Chemical Co., USA) intravenously (dissolved in 0.01 M sodium citrate, pH adjusted to 4.5). This model of experimental diabetes is associated with partial deficits in insulin secretion and consequential hyperglycaemia, without changes in peripheral insulin resistance [10]. STZ-injected animals were accepted as diabetic if blood glucose levels were more than 200 mg/dL [11, 12] using a glucometer (Aquo-Check, Roche) after a one-week period and at least three high blood glucose levels.

 We used three groups randomly constituted four groups: (1) Nondiabetic control animals (C): rats orally fed with standard rat nutrients and water, and (2) diabetic group (D), and (3) rosiglitazone with insulin group (B) treated diabetic animals group (B) rats treated with 4 mg/kg/day RSG two times a day by gavage and Insulin Treatment Protocols eight weeks after the initial STZ injections, diabetic animals were randomly divided into one group. One group of these animals was placed on an insulin regimen (NPH Ilentin II, intermediate acting) for 8 weeks. Insulin doses were individually adjusted so as to maintain euglycemic states and varied between 1 uU/kg (s.c.), given once per day between 9:00 AM. Animals were fed with standard rat nutrient and water without restriction throughout the experiment. Rosiglitazone-treated groups were given group B for 8 weeks and blood glucose levels as well as body weights were measured once weekly.

#### 2.1.1. Isolation of Left Ventricular Muscles

Wistar albino rats were anesthetized with dietyl ether. Hearts were rapidly removed and the left ventricular muscles were dissected. The muscle was mounted in a Petri cup (about 2 mL volume) and perfused continuously (6–8 mL min^−1^) with oxygenated (95% O_2_ and 5% CO_2_) krebs buffer, (constituents in mmol L^−1^: 113 NaCl, 4.7 KCL, 1.2 MgSO_4_·7H_2_O, 1.9 CaCl_2_·2H_2_O, 1.2 KH_2_PO_4_, 25 NaHCO_3_ 11.5 glucose, pH 7,4) solution at a constant flow rate.

### 2.2. Biochemical Analysis

#### 2.2.1. Measurements of HbA1c and Lipid Parameters

Blood plasma HbA1c was determined immunoturbidimetrically (*Pfeiffer* *M*). Triacylglycerol (TAG), total cholesterol (TC), and high-density lipoprotein-cholesterol (HDL-C) were analyzed by glycerophosphate oxidase, peroxidase/4-aminophenazone (GPO/PAP), cholesterol oxidase, peroxidase/4-aminophenazone (CHOD/PAP), and direct COHD/PAP enzymatic colorimetric methods, respectively. The very low density lipoprotein-cholesterol (VLDL-C) and low density lipoprotein-cholesterol (LDL-C) was calculated according to the equation described by Friedewald et al. [13]. All these parameters were determined by Cobas Integra 800 biochemical analyzer (Roche Diagnostics, GmbH, Mannheim, Germany).

#### 2.2.2. Measurement of Malondialdehyde

A tissue specimen of 50 mg was homogenized in 0.15 mol/L KCL. After the homogenate had been centrifuged at 1600 rpm, the MDA levels in tissue homogenate supernatant were determined by the thiobarbituric acid (TBA) reaction according to Yagi [14]. The principle of the method is based on measuring absorbance of the pink color produced by the interaction of TBA with MDA at 530 nm. Values were expressed as nmol/mL.

#### 2.2.3. Measurement of 3-Nitroyrosine

3-NT and tyrosine were obtained from Sigma Chemical (St. Louis, USA). H_2_O_2_, sodium acetate, citrate, NaOH, HCL, H_3_PO_4_, KH_2_PO_4_, and K_2_HPO_4_ were purchased from Merck Chemical (Deisenhofen, Germany). All organic solvents were HPLC grade. The tissues were homogenized in ice-cold phosphate-buffered saline (pH 7.4). Equivalent amounts of each sample were hydrolyzed in 6 N HCI at 100°C for 18–24 h, and then samples were analyzed on an Agilent 1100 series HPLC apparatus (Germany). The analytical column was a 5 *μ*m pore size Spherisorb ODS-2 C_18_ reverse-phase column (4.6 × 250 mm; HICHROM, Waters Spherisorb, UK). The guard column was a C_18_ cartridge (HICHROM, Waters Spherisorb, UK). The mobile phase was 50 mmol/L sodium acetate/50 mmol/L citrate/8% (v/v) methanol, and pH 3.1. HPLC analysis was performed under isocratic conditions at a flow rate of 1 mL min^−1^ and UV detector set at 274 nm. 3-NT and tyrosine peaks were determined according to their retention times and the peaks were confirmed by spiking with added exogenous 3-NT [15] and tyrosine (10 *μ*mol/L). 3-NT levels were expressed as 3-NT/total tyrosine.

### 2.3. Statistical Analysis

The results were expressed as mean ± standard deviation (SD). Kruskal-Wallis (which is nonparametric) test was used for the comparison of groups. When significant differences were observed (*P* < 0.05), Tukey multiple comparison test was used to determine the difference between groups. Statistical analyses were carried out using the SPSS statistical software package (SPSS for Windows version 13.0, SPSS Inc., Chicago, Illinois, USA).

## 3. Results

### 3.1. Effects of Rosiglitazone with Insulin Combination on HbA1c and Lipid Profiles Tolerance in Control and Diabetic Rats

**Figure 1:**
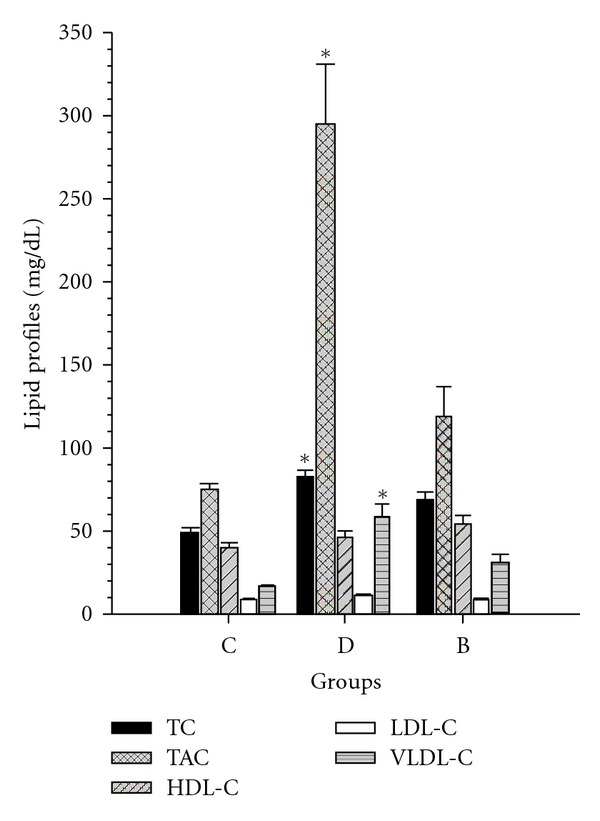
The effects of rosiglitazone with insulin combine on lipid prolifies. C: control rats, D: diabetic rat, group B: rosiglitazone with insulin-treated diabetic rats. Data are expressed as mean + SEM. *P < 0.001 in D compared with group B, C. TAG: triacylglycerol, TC: total cholesterol, HDL-C: high-density lipoprotein-cholesterol VLDL-C; very low-density lipoprotein-cholesterol, LDL-C; low-density lipoprotein-cholesterol.

**Figure 2:**
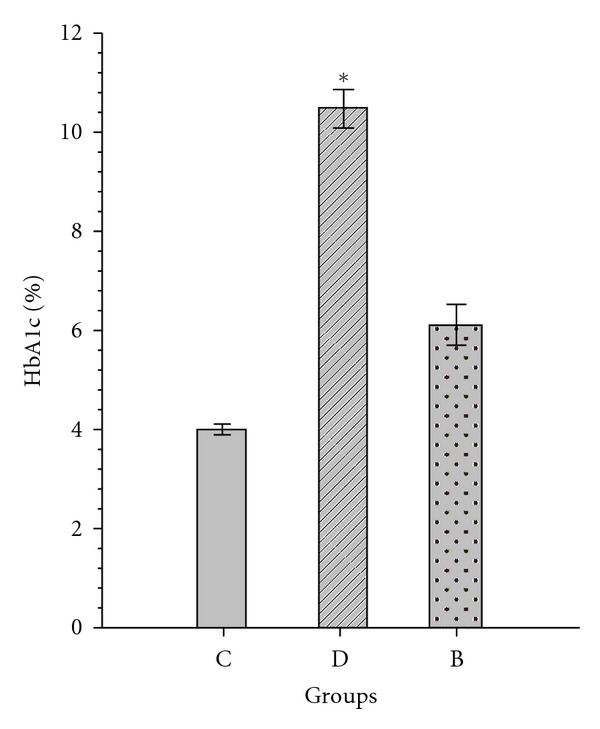
The effects of rosiglitazone with insulin combine on HbA1c. C: control rats, D: diabetic rat, group B: rosiglitazone with insulin-treated diabetic rats. Data are expressed as mean + SEM. *P < 0.001 in D compared with groups B and C. HbA1c: hemoglobin A1c.

TAG: triacylglycerol, TC: total cholesterol, HDL-C: high-density lipoprotein-cholesterol, VLDL-C: very low-density lipoprotein-cholesterol, LDL-C: low-density lipoprotein-cholesterol levels of study groups are shown in Figures 1 and 2. Group B had significant effects on HbA1c and lipid profiles in diabetic rats (*P* < 0.05). HbA1c and TC, TAG and VLDL levels were significantly increased in diabetic group compared with C group (*P* < 0.05). LDL-C levels were not significantly different between groups (Figures 1 and 2).

### 3.2. Effects of Rosiglitazone with Insulin Combination on Blood Glucose in Control and Diabetic Rats

Treatment of D rats with group B resulted in a time-dependent decrease in blood glucose levels. The reduction in blood glucose became significant by week 2 of treatment compared to the diabetic groups (*P* < 0.05) (Table 1). At the end of the study period, the diabetic group had lower body weights than the control group (*P* < 0.05). Treatment of diabetic rats with group B for 8 weeks showed a significant increase (27.3%) in the body weight compared to the diabetic group (*P* < 0.05). On the other hand, D rats for 8 weeks resulted in a significant decrease (39.9%) in the body weight compared to the control group (*P* < 0.05) (Table 2).

### 3.3. Effects of Rosiglitazone with Insulin Combination Therapy on Malondialdehyde Levels in Control and Diabetic Rats

**Figure 3:**
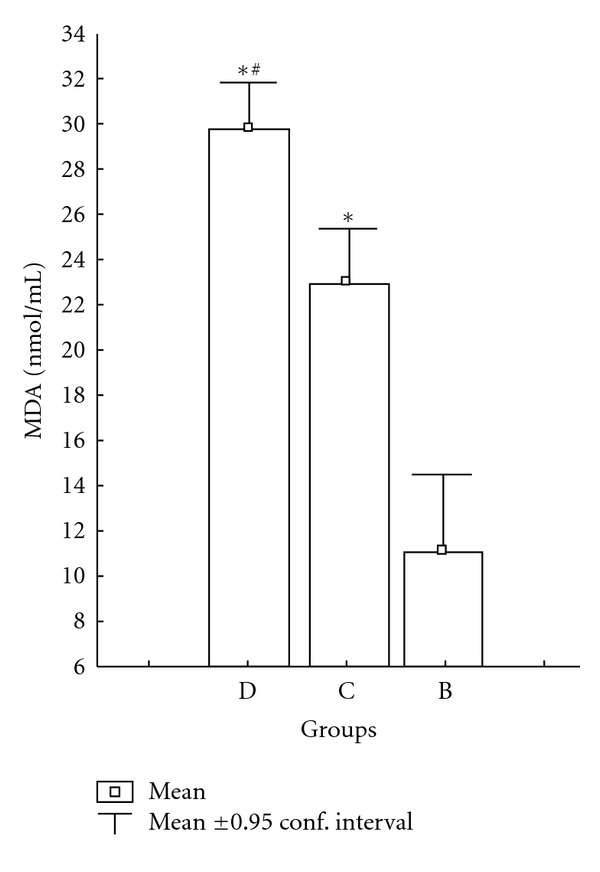
Effects of rosiglitazone with insulin combination therapy on the MDA levels of rat left ventricular. C: control rats, D: diabetic rat, group B: rosiglitazone with insulin combine-treated diabetic rats. Data are presented as mean ± SEM. ^∗#^ *P* < 0.05 in D group compared with C and group B compared with D.

Treatment of diabetic rats with RSG (4 mg/kg/day) for 8 weeks brought about a significant decrease at MDA levels compared with the C groups (*P* < 0.001). MDA levels in D + RSG group were not significantly compared with the B groups. In the diabetic group, MDA levels were found to be increased compared with the C (*P* < 0.04), D + RSG, D + INS, group B (*P* < 0.001) groups and the differences between these groups were significant. MDA levels were not statistically significant between the C and B groups. MDA levels in the C group were significantly different compared to the group B (*P* < 0.003) (Figure 3). 

### 3.4. Effects of Rosiglitazone with Insulin Combination Therapy on 3-Nitroyrosine Levels in Control and Diabetic Rats

**Figure 4:**
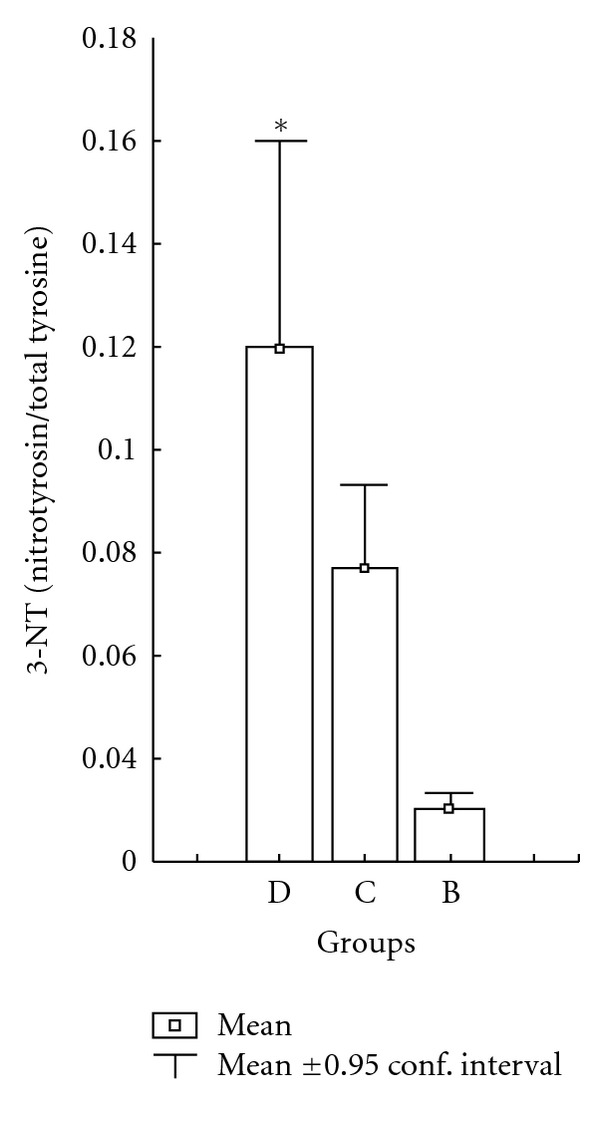
Effects of D + RSG + INS on the 3-nitrotyrosine/total tyrosine levels of rat left ventricular. C: control rats, D: diabetic rat, group B: rosiglitazone with insulin combine-treated diabetic rats. *P < 0.05 in D group compared with groups B and C.

3-NT levels in the C group were not significantly different compared to the D + RSG and D + INS groups. In diabetic group, 3-NT levels were found to be increased when compared with C and B groups and the differences between these groups were significant, respectively (*P* < 0.005). There were no statistically significant differences between the B groups. Treatment of diabetic rats with group B (4 mg/kg/day and 1 uU/kg^−1^) for 8 weeks brought about a significant decrease at 3-NT levels compared with the C and B groups (*P* < 0.0011). There were no statistically significant differences between the B groups (Figure 4).

## 4. Discussion

In this study, we have demonstrated that group B prevents glucose-induced oxidative stress in cardiac cells, an effect independent from PPAR*γ*, but distinctively dependent on activated protein kinase (AMPK) activation. We also showed that the ability of RSG to quench oxidative stress is conveyed through the inhibition of NADPH oxidase. Furthermore, we demonstrated that, downstream of AMPK activation, the effect of RSG + INS on glucose-induced NADPH oxidase-derived ROS production is mediated by the inhibition of the diacylglycerol (DAG)-*protein* *kinase  C  *(PKC) pathway.

In this study, we investigated the effect of RSG, a member of the TZD family, on lipid profile and oxidative status in STZ-induced diabetes mellitus rats. Many studies have reported that TZDs act through PPAR*γ*-dependent mechanisms, and this is also true in endothelial cells. For instance, RSG increased NO production in human umbilical vein endothelial cells through a transcriptional mechanism unrelated to eNOS expression but dependent on PPAR*γ* activation [16]. Interestingly, this effect has been attributed to the inhibition of NO quenching by NADPH oxidase-derived ROS [17].

Rosiglitazone is an agonist of the PPAR-*γ*, which is found in insulin-dependent glucose-requiring tissues such as adipose tissue, skeletal muscle, left ventricular muscle, and liver tissue [18, 19]. The end result of PPAR-*γ* activation is a reduction in hepatic glucose production and increased insulin dependent glucose uptake in fat and skeletal tissues [8, 19, 20].

Calkin et al. [21] observed that RSG had a significant effect on HbA1c in diabetic mice. In addition, previous studies have reported that the administration of RSG induced a significant decrease in serum glucose levels [18, 22]. In our study, in group B-treated diabetic rats mean blood glucose significantly decreased rapidly from 335 to 202.1 mg/dL between weeks 0 and 8. The blood glucose levels started to be reduced at 2th week of treatment compared to the level of diabetic groups. Malinowski and Bolesta [23] found that treatment of RSG responses began to be observed at 4th week and were maximal at 12th week. With regard to this report, RSG may indicate its full effect on blood glucose at 12th week. In our previous studies, it was indicated that the effects of treatment with RSG on the body weight of diabetic rats was significant [18], but treatment of diabetic rats with RSG (4 mg/kg/day) was caused a significant increase compared with diabetic rats by the end of the treatment period [24]. In our study, statistically significant increases in body weight were observed group B-treated diabetic rats. These weight changes may be function increased adipocyte differentiation, which is one of the primary effects of group B. The clinical significance of these modest weight changes will require further evaluation in a long-term study.

We found a graded and significant elevation of glucose levels from the initial phase through the sacrifice in D group with and without RSG and we believe that it could be secondary to the development of some degree of insulin resistance although this was not evaluated in the present study. The insulin-resistant state is commonly associated with lipoprotein abnormalities such as hypertriglyceridemia, high levels of VLDL, small dense LDL [25], and low levels of HDL-cholesterol [26], which are risk factors for coronary heart disease. The hypoglycemic and hypotriglyceridemic action of TZDs is through the activation of PPAR-gama leading to increased insulin sensitivity of peripheral tissues and lipoprotein lipase activity in the adipose tissue [27]. Zhao-hui et al. [28] did not show a reduction of blood glucose level in hypercholesterolemic rabbits receiving RSG for 6 weeks, as in the present study. Furthermore, we also observed a significant elevation of triglycerides and HDL-C at the time of euthanasia in RSG group. The effects of TZDs on triglycerides have been somewhat more variable. Decreases in triglyceride levels have been more frequently observed with pioglitazone than with rosiglitazone. We cannot rule out that these effects on glucose and triglycerides were due to chance, as our evaluation period was short and the sample was relatively. These findings are quite controversial in the literature [28–30].

Boyle et al. [31] found that RSG reduced TAG, but increased total cholesterol, LDL-C, and reduced HDL-C. In contrast, pioglitazone reduced TAG, total cholesterol, and LDL-C and increased HDL-C. Conversely, in their study, Myerson et al. [32] observed reduced plasma fatty acid concentrations and hepatic TAG content after RSG therapy. Within that TZD group, marked differences have been reported as regards the effect of different members on lipid profiles in patients with type-2 diabetes. In the present study, the administration of RSG + INS, a member of the TZD family, decreased TC, TAG, and VLDL-C, LDL-C levels in STZ diabetic rat.

 Bagi et al. [33] reported that the reduced activity of catalase may result in enhanced hydroxyl radical production leading to enhanced lipid peroxidation in diabetes. Even short-term activation of PPAR-*γ* by RSG reduces NAD(P)H oxidase and enhances catalase activity causing a reduction of superoxide and hydroxyl radical production, thereby enhancing NO mediation of coronary vasodilation and reducing lipid peroxidation in diabetes. These findings suggest that activation of PPAR-gamma may exert an antioxidant activity by favorably altering the expression of specific enzymes participating in the production and/or elimination of reactive oxygen species.

 Radi et al. [34] found that elevated levels of MDA were brought down to the normal values by treatment with RSG. In the present study, we have found that compared with controls, in diabetic rats, the serum level of MDA (a marker of in vivo lipid peroxidation) was significantly elevated, which was reduced by group B treatment (Figure 3). On the basis of study findings, we observed that even short-term RSG treatment of rats with diabetes would, by reducing oxidative stress. These results suggest that RSG + INS is capable of reducing oxidative stress in rats with diabetes. Patients with diabetes have modified levels of various markers of oxidative stress, indicating an overproduction of free radicals, which have a key role in the development of diabetic vascular complications [35]. When focusing on diabetic vascular disease, it is the fine balance between the levels of superoxide (O_2_^−^), peroxynitrite (ONOO^−^), and NO, that is, the key in determining the extent of vascular damage. It is noteworthy that TZD show intracellular antioxidant activity. This property may reflect “preventative” action since these agents do not show direct antioxidant scavenging activity on free radicals, but block several mechanisms that in hyperglycaemic or hyperlipidaemic conditions lead to the generation of oxidative stress. It has been observed that PPAR-*γ* ligands inhibit the expression of inducible NO synthase (iNOS) and, consequently, ONOO^−^ production, in mesangial cells and in cerebellar granule cells [36]. Similarly, in mice with rheumatoid arthritis, pioglitazone and RSG have been shown to reduce the expression of iNOS and nitrotyrosine deposition [37].

It seems that this is the first study to investigate the effect of group B on oxidative/nitrosative effect in left ventricular of diabetes mellitus rats.

In summary, the present study demonstrates that in diabetes, treatment with rosiglitazone with insulin combined caused rapid reductions in oxidative stress that are not associated with corrections of major metabolic derangements. Our results clarify that mechanisms of TZD-induced vascular protection include suppression of specific NADPH oxidase subunit expression and ventricular muscle superoxide production similar to previously reported direct effects of PPAR*γ* ligands on vascular endothelial cells in vitro [38]. The mechanisms of PPAR*γ*-induced suppression of NADPH oxidase subunits remain to be defined and constitute an area of active investigation in our lab. We also postulate that improvements in endothelial dysfunction caused by sustained PPAR*γ* activation in diabetes as well as in other disorders associated with endothelial dysfunction may be related to direct effects of PPAR*γ* activation on endothelial nitric oxide synthase activity [39] mediated by TZD-induced alterations in post-translational mechanisms regulating eNOS activity [40].

## 5. Conclusions

Ongoing studies in our laboratory will determine if direct TZD-mediated activation of PPAR*γ* coordinately regulates the production of O^2−^ and nitric oxide at the level of the vascular wall to modulate the development of endothelial dysfunction. These studies could further clarify the vascular protective effects of PPAR*γ* ligands and Thus, in diabetes-related vascular diseases RSG treatment may be cardioprotective.

## Figures and Tables

**Table 1:** Blood glucose levels in the study groups.

Weeks									
Groups	0	1	2	3	4	5	6	7	8
C (mg/dL)	99.1 ± 1.1	101.2 ± 1.9	102.0 ± 1.7	99.6 ± 1.7	99.1 ± 1.2	98.8 ± 1.7	99.4 ± 1.4	101.1 ± 2.7	100.7 ± 1.6
D (mg/dL)	102.5 ± 1.8	275.8 ± ^∗^10.0	301.9 ± ^∗^4.8	306.9 ± ^∗^10.1	318.5 ± ^∗^16.3	319.6 ± ^∗^11.8	321.5 ± ^∗^9.3	322.2 ± ^∗^9.7	335.7 ± ^∗^16.7
B (mg/dL)	92.2 ± 3.1	306.3 ± 7.8	273.5 ± ^†^22.7	272.2 ± ^†^20.1	262.9 ± ^†^18.5	261.5 ± ^†^28.2	169.0 ± ^†^29.1	200.3 ± ^†^12.9	202.1 ± ^†^6.4

C: control rats, D: diabetic rat, B: rosiglitazone with insulin combine-treated diabetic rats. Data are presented as mean ± SEM. ^†^ *P* < 0.05 in B groups compared with D; ^∗^ *P* < 0.05 in D compared with C in the same group in the same week.

**Table 2:** Body weight levels in the study groups.

Weeks									
Groups	0	1	2	3	4	5	6	7	8
C (gr)	100.0 ± 0.8	100.8 ± 0.8	101.1 ± 0.7	102.7 ± 0.7	107.6 ± 2.2	107.5 ± 0.8	109.3 ± 0.7	111.2 ± 0.8	112.6 ± 0.9
D (gr)	100.0 ± 1.8	91.5 ± 1.3	86.8^∗^ ± 1.3	75.5^∗^ ± 2.8	75.0^∗^ ± 1.7	73.5^∗^ ± 1.5	70.9^∗^ ± 1.5	71.6^∗^ ± 1.6	72.7^∗^ ± 1.8
B (gr)	100.0 ± 2.0	94.0 ± 2.1	89.1 ± 1.4	91.0^†^ ± 1.2	95.3^†^ ± 2.5	96.0^†^ ± 2.4	97.0^†^ ± 1.8	99^†^ ± 2.3	100.0^†^ ± 2.1

C: control rats, D: diabetic rat, B: rosiglitazone with insulin combine-treated diabetic rats. Data are presented as mean ± SEM. ^†^ *P* < 0.05 in B groups compared with D; ^∗^ *P* < 0.05 in D compared with C in the same group in the same week.
